# Cardiovascular Risk Factors in Bulgarian Patients with Polycystic Ovary Syndrome and/or Obesity

**DOI:** 10.1155/2012/306347

**Published:** 2012-01-04

**Authors:** Antoaneta Gateva, Zdravko Kamenov

**Affiliations:** Clinic of Endocrinology, University Hospital Alexandrovska, Medical University-Sofia, 1 Georgi Sofiiski Boulevard, 1431 Sofia, Bulgaria

## Abstract

Polycystic ovarian syndrome (PCOS) is one of the most common endocrine disturbances in women of reproductive age. Besides its well-known effects on reproductive health, it is also linked to increased cardiovascular risk in later life. The aim of this study is to investigate some classical cardiovascular risk factors in a crossectional study of Bulgarian women with PCOS and/or obesity. We performed a retrospective medical chart review of 375 women from an university endocrine clinic. We found significant differences in the indices of carbohydrate metabolism, blood pressure, lipid profile, rate of liver steatosis, and the levels liver enzymes and hematological results between the lean and obese PCOS women. Obese women without PCOS did not show significantly different results in their OGGT form obese PCOS women. Waist-to-stature-ratio (WSR) correlated better with the baseline IRI levels and lipid profile than waist-to-hip-ratio (WHR) that makes it a better marker for unfavorable metabolic profile.

## 1. Introduction

PCOS is a prevalent disorder that affects approximately 6–10% of women of reproductive age [1, 2] and is a major cause of menstrual disturbances, hirsutism, and female anovulatory infertility. Many PCOS women show evidence of insulin resistance and hyperandrogenism. Current evidence suggests that insulin resistance and compensatory hyperinsulinemia are a central feature of PCOS [3]. Hyperinsulinemia appears to play an important pathogenic role in the hyperandrogenism of both obese and lean women with PCOS [4–6]. According to some studies, PCOS patients have an increased risk for diabetes mellitus [7, 8] and often show an adverse cardiovascular risk profile-increased rate of arterial hypertension [9, 10], dislipidemia [10–14], and subclinical inflammation and atherosclerosis [15–17]. Cardiovascular risk factors are usually present even in younger age and this suggests that the chronic disturbances in hormonal and metabolic status typical for the syndrome predispose the patients to development of early atherosclerosis and premature clinical presentation of cardiovascular disease.

Obesity plays an important role in the pathogenesis of PCOS, and around 30–75% of PCOS women are obese [18–20]. Over the last 20 years, the prevalence of obesity has dramatically increased, with probable associated increase in PCOS. Obesity contributes to the prevalence of the metabolic syndrome in PCOS patients [21]. Central obesity is often associated with PCOS [22] and carries increased risk for developing cardiovascular disease and type 2 diabetes [23]. The specific indicators of abdominal obesity are better for discriminating the high coronary risk than the usual obesity indicator BMI (body mass index) [24]. In clinical practice waist-to-hip ratio (WHR) has been used to determine the presence of central obesity. There are some indications however that waist-to-stature ratio (WSR) or waist circumference may predict cardiovascular risk better than BMI or WHR [25]. WSR has not been thoroughly studied as a predictor of cardiovascular risk in patients with PCOS. The aim of this study is to investigate some classical cardiovascular risk factors in a crossectional study of Bulgarian women with PCOS and/or obesity.

## 2. Materials and Methods

The sources of information in this study were the available medical charts of the patients referred to the Clinic of Endocrinology in Alexandrovska University Hospital-Sofia for the last 21 years (starting year 1990). In this retrospective study were included patients with diagnosed PCOS or obesity that had sufficient data in their medical charts to be included in the analysis.

Electronic database was created that included the following information for each patient.

1. *General Information*: name, age, place of residence, date of admittance, hospital stay.
2. *Anthropometric Data*: height, weight, body mass index (BMI), waist circumference, hip circumference, waist-to-hip ratio (WHR), waist-to-stature ratio (WSR).
3. *Obesity* was accepted at BMI ≥ 30 kg/m^2^.
4. *Polycystic Ovary Syndrome* was diagnosed according to the ESHRE-ASRM criteria—two out of the following: (1) oligo/amenorrhea; (2) clinical or biochemical hyperandrogenism; (3) polycystic ovaries at ultrasound examination when all other endocrine causes are excluded.
5. *Arterial Hypertension* was accepted if there was such a diagnosis in the file and/or there was antihypertensive treatment and/or measured during the hospital stay arterial blood pressure ≥140/90 mmHg. The duration of the hypertension, present blood pressure—systolic (SBP) and diastolic (DBP) number and type of antihypertensive medications were also recorded
6. *Lipid Profile*: total cholesterol (TC), HDL-cholesterol (HDL), LDL-cholesterol (LDL), VLDL-cholesterol, triglycerides (TG). *Dislipidemia* was accepted if there was such a diagnosis in the file and/or there was any treatment for dislipidemia and/or measured during the hospital stay TC > 5.2 mmol/L, and/or HDL < 1.3 mmol/L and/or TG > 1.8 mmol/L. The duration of the dislipidemia, present lipid profile, type of treatment and number of medications were also recorded.
7. *Gynecological History*: number of pregnancies and births, miscarriages, age of menarche, duration of the menstrual cycle, menstrual disturbances (amenorrhea, oligomenorrhea, hypermenorrhea, polymenorrhea, opsomenorrhea). Regular menstrual cycle was defined as a menstrual cycle between 21 and 35 days and with intercycle variability 2-3 days. Oligomenorrhea was defined as a menstrual cycle longer than 35 days and shorter than 6 months. Amenorrhea was defined as a menstrual cycle longer than 6 months.
8. Data about the presence of *Clinical Hyperandrogenism* (hirsutism, acne, androgenic alopecia).
9. Results form the *Oral glucose Tolerance Test* (OGTT): blood glucose and imunoreactive insulin (IRI) on 0, 60, and 120 min.
10. *Hormonal Status* (testosterone, androstendione, dehydroepiandrosteron sulphate (DHEAS), 17-OH-progesterone, estradiol, LH, FSH, TSH, cortisol rhythm, prolactin, etc.).
11. *Liver Enzymes*: ASAT, ALAT, GGT, AP
12. *Hematological Status* (after exclusion of actual acute inflammation): hemoglobin, hematocrit, red blood cells (RBC), white blood cells (WBC), mean corpuscular volume (MCV), mean corpuscular hemoglobin (MCH), mean corpuscular hemoglobin concentration (MCHC), mean platelet volume (MPV), erythrocyte sedimentation rate (ESR).
13. *Ultrasound Data* for liver steatosis (fatty liver disease).
14. *Gynecological Ultrasound Data*: right and left ovary measurements and volume, presence of polycystic ovaries, size of the uterus, endometrium.
15. *ECG Results*: heart rate, QT-interval, RR-interval, presence of ischemia.
16. Data about *Medication Use*: metformin or combined oral contraceptives (COC)—type and dose.
17. Data about *Ovarian Surgery*: wedge resection or endoscopic ovarian drilling.

All laboratory tests were performed in the Central Clinical Laboratory of the Alexandrovska University Hospital in Sofia, which is the reference laboratory for the country. Because in the different periods included in the retrospective study there were some differences in the refference ranges, we used the following formula to unify the data:

(1) $$Y=a_{0}+\frac{\Delta a\left(x-b_{0}\right)}{\Delta b},$$

where *Y* is the standard value; *a*_0_ is the lower limit of the standard reference range, *b*_0_ is the lower limit of the given scale, Δ*a* is the difference between the upper and the lower limit of the standard reference range.

Hormones from the gonadal axis were measured during the early follicular phase after spontaneous or progestin induced bleeding.

For *standard reference range* of the hormones were accepted the following:

Testosterone—0.3–3.0 nmol/L,

Androstendione—0.73–10.7 nmol/L,

17-OH-progesterone—0.45–3.3 nmol/L,

DHEAS—1.65–11 mcmol/L,

Estradiol—0.08–0.79 pmol/L,

LH—0.7–9.0 mU/L,

FSH—0.6–9.5 mU/L,

Prolactin—50–659 mU/L,

Cortisol 8 o'clock—171–536 nmol/L,

Cortisol 22 o'clock—64–340 nmol/L,

TSH—0.2–4.2 mU/L.

In the study, were included premenopuasal women with obesity and/or PCOS. Exclusion criteria were considered as age less than 18 or higher than 45 years, postmenopausal state, severe hepatic, cardiovascular or endocrine disorders (incl. diabetes), or other concurrent medical illnesses. The patients were divided into three groups—group 1 Obese; group 2 Lean PCOS, and group 3 Obese PCOS. Comparison was made between the data from these three groups.

As a part of this retrospective study, we performed an analysis of the data from the OGTT using different criteria for insulin resistance:

1. elevated baseline IRI,
2. decreased baseline glucose/insulin ratio (less than 0.333 mmol/L/mU/L),
3. increase of the level of the IRI more than 100 mU/L during the OGTT,
4. increase of the level of the IRI more than 5 times from the baseline,
5. HOMA index more than 2.0, calculated as baseline blood glucose (mmol/L) multiplied by baseline IRI (mU/L) and then devided by 22.5.

For this part of the study were used only the patients that had the full data from the OGTT (blood glucose on 0,60 and 120 min and IRI on 0, 60, and 120 min). For every patient were examined the presence of insulin resistance by each of these criteria and its prevalence in the general group and separately in each subgroup.

### 2.1. Statistical Methods

The data were processed using the statistical package SPSS 16.0. The level of significance for rejecting the null hypothesis was *P* < 0.05. The following statistical methods were applied: descriptive analysis, variation analysis, Kolmogorov-Smirnov's one sample nonparametric test, Student's *t*-test for two independent samples, Kruskal-Wallis' nonparametric test for several independent samples, Mann-Whitney's nonparametric test for two independent samples, one-way analysis of variance between-groups ANOVA with post hoc tests, and correlation analysis.

## 3. Results

Initially the present study included 375 women. After age-matching of the three groups, 74 women with obesity were excluded and at the end 301 women aged 18 to 40 years participated in the analysis. Patients' characteristics are shown in Table 1.

Obese PCOS women did not significantly differ in weight, BMI, waist, hip, WHR, or WSR from the obese women without PCOS.

The results from the comparison between the three groups are shown on Tables 2–7. A statically significant difference was found regarding the results from the OGTT (blood glucose on 0 and 60 min and IRI on 0, 60, and 120 min), HOMA-index, glucose/insulin ratio, the rate of hypertension and dislipidemia, systolic and diastolic blood pressure, HDL, VLDL, and TG (Table 2), the rate of liver steatosis and the levels of ALAT, GGT, and AP, and hematological results (WBC and ESR) (Table 3) between the lean and obese PCOS women.

Obese women without PCOS did not show significantly different results in their OGTT form obese PCOS women. These two groups however differ in the levels of testosterone and LH, ovarian volume (Table 4) and the rate of hirsutism (Table 6) and menstrual disturbances (length of menstrual cycle and the prevalence of oligomenorrhea).

A strong positive correlation was found between the body weight and baseline IRI and HOMA index in general (*r* = 0.58 and *r* = 0.59, resp., *P* < 0.01) that is most prominent in lean PCOS group. The correlation is weaker for IRI on 60 and 120 min of OGTT (*r* = 0.25 and *r* = 0.14, resp., *P* < 0.01). It is worth mentioning that WSR correlates better with the baseline IRI levels and HOMA-index (*r* = 0.31 and *r* = 0.59, resp., *P* < 0.05) than WHR (*r* = 0.18 and *r* = 0.38, resp., *P* < 0.05) that makes it a better marker for unfavorable metabolic profile.

A weak but statistically significant correlation was observed in the total group between the results from the OGTT (except fasting blood glucose) and systolic and diastolic blood pressure that disappear when patients are divided into groups according to diagnosis, except for the intermediate correlation that exists between SBP, DBP, and blood glucose on 120 min (*r* = 0.263 and *r* = 0.224, *P* < 0.05) IRI on 120 min (*r* = 0.438 and *r* = 0.428, *P* < 0.05) only in obese PCOS patients. Again, WSR correlates better with SBP, DBP, and the rate of hypertension than WHR (*r* = 0.286, *r* = 0.263, and *r* = 0.198, resp., versus *r* = 0.136, *r* = 0.135, and *r* = 0.032, resp., *P* < 0.05).

Differences in HDL-cholesterol, VLDL-cholesterol, and triglycerides were found between the lean PCOS patients and the other two groups. There is an intermediate correlation between the body weight and the levels of triglycerides (*r* = 0.39, *P* < 0.01) that after dividing the patients into groups persisted only in the lean PCOS group. Only in these patients, we observe a negative correlation between HDL-cholesterol and body weight (*r* = − 0.45, *P* < 0.01). The same as for carbohydrate metabolism, WSR also shows stronger correlation than WHR with the levels of HDL (*r* = − 0.27 versus *r* = −0.10), VLDL-choleserol (*r* = 0.38 versus *r* = 0.29, *P* < 0.05), and TG (*r* = 0.41 versus *r* = 0.27, *P* < 0.05) but not with TC (*r* = 0.03 versus *r* = 0.14).

As for liver function, the levels of the liver enzymes and the rate of liver stetaosis are significantly lower in the lean PCOS patients. A strong correlation between the body weight and the presence of liver steatosis (*r* = 0.56, *P* < 0.01) was found so we can say that the main factor for development of nonalcoholic fatty liver disease is the obesity. The levels of GGT but not ASAT, ALAT, or AP correlate with IRI on 0 and 60 min and HOMA index. The rate of liver steatosis also shows good correlation with IRI during the OGTT, HOMA index, glucose/insulin ratio, and diastolic blood pressure.

WBC showed correlation with baseline blood glucose and HOMA index (*r* = 0.340 and 0.410; *P* < 0.05) but not with the results during OGTT. There is also intermediate correlation between WBC and VLDL and TG-levels (*r* = 0.358 and 0.333, resp., *P* < 0.01). On the other hand, ESR also shows good correlation to HOMA index, BMI, WHR, and WSR (*r* = 0.360; *r* = 0.441; *r* = 0.242; *r* = 0.397; *P* < 0.01) and not to any of the androgen levels.

The levels of testosterone and androstendione did not show significant correlation to the results of OGTT, lipid levels, blood pressure, liver enzymes, or blood count in the total group. Hemoglobin and hematocirte show a weak positive correlation to the testosterone levels (*r* = 0.239 and *r* = 0.199) but not to the levels of other androgens.

Interestingly, obese PCOS patients have significantly higher rate of family history for coronary heart disease compared to the other two groups (Table 8). Lean PCOS patients have higher rate of family history of PCOS than obese PCOS patients. On the other hand, obese patients with or without PCOS have higher rate of family history for diabetes mellitus, hypertension, and obesity than lean patients.

As expected, the main reason for admittance of the obese patients without PCOS is obesity per se while for the other two groups the leading reason are menstrual disturbances followed by hirsutism. However, menstrual disturbances as a reason for admittance are significantly more common in lean PCOS patients than in obese PCOS patients.

The use of metformin is most frequent in the obese PCOS group and does not significantly differ between the other two groups (Table 7). That can be explained by the more prominent insulin resistance in patients that have both obesity and PCOS. It must be noted, however, that lean PCOS patients start insulin sensitizing therapy as frequent as obese women without the syndrome. There still remains opened the question how is insulin resistance detected in every single patient (Table 8).

Of 375 women in our study 155 had the full set of data from the OGTT (blood glucose and IRI on 0, 60, and 120 min). In every one of them, we assessed the presence or the lack of impaired fasting glucose, glucose tolerance, or insulin resistance according to these criteria.

The presence of insulin resistance using the increased baseline insulin or insulin elevation over 100 mU/L during the OGTT correlates better with the arterial blood pressure and lipid levels than when using elevation of IRI more than 5 times over the baseline (Table 9).

These data show a lower level of insulin resistance in lean PCOS subjects compared to the other groups. There is also a different rate of insulin resistance when using different criteria in the total group and in every subgroup. In the three groups, there is a higher rate of insulin resistance when using the elevation of IRI more than 5 times over the baseline. In this case, the rate of insulin resistance in the three groups is similar. It should be noted, however, that the highest elevation of IRI is seen in some patients on the 60 min of OGTT and in others on 120 min of the OGTT. For this reason to benefit from the full diagnostic power of the method, it is necessary to measure IRI both on 60 and 120 min, otherwise, we would miss patients with insulin resistance.

## 4. Discussion

The aim of the study was to investigate some cardiovascular risk factors in young women with PCOS. This is the largest study on PCOS patients in Bulgaria.

The link between the obesity and reproductive problems in women has been studied for a long time and is confirmed from the results of a number of epidemiological and clinical studies (for review [26]). The rate of obesity in patients with PCOS is between 30% and 70% depending on the ethnicity and the used criteria for diagnosis of PCOS [27, 28]. Therefore, PCOS patients can be divided into two main phenotypes according to their BMI-lean and obese that have very different metabolic and cardiovascular risk profile. Although the insulin resistance is thought to be fundamental for PCOS, obesity tends to worsen it and the metabolic disturbances [29]. Our data support this theory and show that the indices of carbohydrate metabolism—fasting blood glucose and blood glucose on 60 min of OGTT and IRI on 0, 60, and 120 min of OGTT and HOMA index are significantly lower in lean PCOS subjects but do not differ significantly in obese and obese PCOS women although obese PCOS patients have higher mean values. This is why it can be postulated that the obesity and PCOS worsen their unwanted effects on the carbohydrate metabolism and lean PCOS women have comparatively discrete disturbances.

Our data show that the metabolic indices, SBP, DBP, and hematological results are highly dependent on the presence of obesity and not on the presence of PCOS in the patients. The hyperinsulinemia has an important role in the development of hypertension by increasing the sodium retention [30], and in this way, it leads to increased intracellular levels of sodium and calcium [31]. Insulin also increases the release of IGF-1, which can lead to hypertension as a result of nonstriated-muscle hypertrophy. We found a correlation between IRI during OGTT and systolic and diastolic blood pressure.

Many patients with PCOS have classical signs of metabolic syndrome. In healthy women with normal body weight and preserved insulin sensitivity, the adipocytes release small amounts of free fatty acids (FFAs) and have a normal activity of the lipoprotein lipase (LPL). In these women, the physiological levels of testosterone act in synchrony with the insulin and suppress the release of FFA, so they have antilipolytic effect [32]. In women with obesity, there is an increased production of FFA and decreased activity of LPL as a result of the prominent insulin resistance. In these conditions, the high androgen levels additionally worsen the disturbances in the lipid metabolism [32]. It is thought that approximately 70% of the patients with PCOS have disturbances in serum lipid levels [33]. Even after weight adjustment, the lipid abnormalities persist [10, 34]. In our study, we found differences in HDL, VLDL, and TG between the lean PCOS patients and the other two groups.

There are data that the total white blood cell count is an independent risk factor for coronary heart disease [35, 36] and related to it morbidity and mortality [37]. In some studies in women with PCOS, there are higher WBC levels than in healthy women that correlate well with the markers of insulin resistance (HOMA index) [38]. We also found correlation between WBC and VLDL and TG-levels that promote the development of atherosclerosis. We found significantly higher levels of ESR in obese compared to lean PCOS patients. ESR showed positive correlation to BMI, WHR, WSR, and HOMA index. To our knowledge, this is the first study that assesses the ESR in PCOS patients. The increased levels of white blood cells and ESR are probably linked to subclinical inflammation that plays an important role in early clinical manifestation of atherosclerosis.

Testosterone stimulates the hematopoiesis in the bone marrow and, consequently, increases the hematocrit [39]. Hypogonadal men have a statistically significant lower hematocrit [40]. In accordance to this, we found a weak correlation between the levels of testosterone but not with other androgens to hemoglobin and hematocrit levels. In our study, lean and obese PCOS patients do not significantly differ in their testosterone levels and that is why they have similar blood count indices. On the other hand, obese PCOS patients have higher testosterone than obese women without PCOS and that goes with significantly higher hemoglobin but not hematocrit. It is important to note that hemoglobin and hematocrit correlate with testosterone but not with other androgen levels.

The indices of clinical hyperandrogenism are the highest in the obese PCOS patients and this confirms the theory that obesity worsens the disturbances caused by PCOS and aggravates the clinical presentation of the syndrome.

The family history for obesity is more frequent in obese patients no mater if they have PCOS or not. On the other hand, the family history for PCOS is more frequent in lean PCOS women and this suggests the more important role of the genetic milieu in these cases. Unlike that for obese PCOS women, probably of utmost importance is the presence of obesity. Studies show higher prevalence of diabetes mellitus in relatives of PCOS patients [41, 42] and particularly of those with impaired glucose tolerance or overt diabetes [43]. We found a higher rate of family history for diabetes only in obese PCOS patients. These patients also have notably higher family history of coronary heart disease that can be related to the increased cardiovascular risk in later life.

The absolute waist circumference (>88 cm) and the WHR (>0.85) are both used as measures of central obesity in women [44]. Recently, another measure of central obesity was proposed which has shown superiority to BMI in predicting cardiovascular disease risk is the waist-to-stature ratio (WSR), where a ratio of ≥0.5 (i.e., a waist circumference at least half of the individual's height) is predictive of increased risk [45]. In light of concerns raised about the ability of BMI alone to predict cardiovascular risk, some studies have recently attempted to compare BMI with waist circumference and other anthropometric measures of obesity, such as WHR and WSR, as predictors for cardiovascular risk. In a meta-analysis of abdominal obesity indices comparing BMI, waist circumference, WHR, and WSR, researchers concluded that WSR was the best predictor for both hypertension and dislipidemia for both men and women, while BMI was the least accurate predictor of hypertension [46]. To our knowledge, our study is the first to show that WSR is a better marker for unfavorable metabolic profile (OGTT results, blood pressure, and lipid profile) than WHR in a specific group of PCOS patients. Further large-scale studies are necessary to position the different anthropometric parameters, according to definite endpoints.

Our results confirm the presence of insulin resistance both in lean and obese PCOS women that is, however, more pronounced in obese subjects. According to our data, the increased baseline insulin is much more sensitive in patients with obesity with or without PCOS than the standard OGTT without IRI measurement regarding the carbohydrate metabolism disturbances. This is not seen in lean PCOS subjects where the rate of IFG/IGT is the same as that of increased baseline insulin. In our previous study, it was shown that IGT and/or diabetes on 120 min of OGTT was diagnosed in 8.5% of the women with PCOS while IFG and/or diabetes on 0 min of OGGT—only in 2.2% of the patients [47]. In the present study, however, patients with diabetes were excluded, but in the whole population with OGTT, 12.9% had disglycemia—IFG and/or IGT.

In the three groups, insulin resistance according to the criteria of IRI over 100 mU/L during the OGTT is seen two times more frequently on 60 min than on 120 min. So, if for some reason only two measurements of IRI during the OGTT are available, they must be on 0 and 60 min and not on 0 and 120 min. This is mostly true for lean PCOS patients because in the other two groups the diagnostic powers of the increased baseline insulin levels are comparable to the response of the insulin on 60 min and exceed that on the 120 min.

As to the HOMA index, its ability to diagnose the insulin resistance is highest in obese PCOS patients and lowest in lean PCOS patients. That is true also for decreased glucose/insulin ratio.

**Limitations**

Our study has all the limitations of retrospective crossectional studies—lack of all data for each patient, different clinical approach during the different periods, and so forth.

## 5. Conclusion

1. Obesity is the most important factor for unfavorable metabolic and cardiovascular risk profile in PCOS patients.
2. In our study population, WSR is a better anthropometric marker of adverse metabolic profile in women with PCOS and/or obesity than WHR.
3. Carbohydrate metabolism testing is necessary in all PCOS patients because of the high prevalence of its disturbances.
4. In patients with obesity with or without PCOS only the baseline indices-glucose and IRI and HOMA index can be used.
5. In lean PCOS patients, an OGTT with IRI measurement on 0, 60, and 120 min is highly recommended.
6. ESR show positive correlation with BMI and the indices of insulin resistance, but not with androgen levels.

## Figures and Tables

**Table 1:** Anthropometric characteristics of the groups.

	Group 1 Obese (*n* = 125)	Group 2 Lean PCOS (*n* = 94)	Group 3 Obese PCOS (*n* = 82)
Age (years)	26.50 ± 5.47	25.17 ± 4.64	26.26 ± 5.64
Height (cm)	162.99 ± 6.09	163.39 ± 6.52	163.68 ± 6.70
Weight (kg)	99.82 ± 17.00^∧∧∧^	61.56 ± 10.32***	97.09 ± 14.07
BMI (kg/m^2^)	37.55 ± 5.95^∧∧∧^	23.04 ± 3.47***	36.21 ± 4.58
Waist (cm)	106.92 ± 12.49^∧∧∧^	77.81 ± 9.87***	103.69 ± 10.24
Hip (cm)	121.78 ± 11.70^∧∧∧^	96.93 ± 8.34***	118.92 ± 9.39
WHR	0.86 ± 0.08^∧∧∧^	0.79 ± 0.06***	0.87 ± 0.08
WSR	0.65 ± 0.08^∧∧∧^	0.47 ± 0.06***	0.63 ± 0.07

***P < 0.001  between group 2 and group 3.

^*∧∧∧*^ *P* < 0.001 between group 1 and group 2.

**Table 2:** Comparison between the results from oral glucose tolerance test, lipid profile, blood pressure measurement, and ECG characteristics between the three groups.

	Group 1		Group 2		Group 3	
	Obese		Lean PCOS		Obese PCOS	
	*n*		*n*		*n*	
Fasting blood glucose (mmol/L)	125	4.37 ± 1.02	94	4.25 ± 0.75*	82	4.63 ± 0.94
Blood glucose on 60 min of OGTT (mmol/L)	110	7.62 ± 1.99^∧^	75	6.72 ± 2.08**	78	7.87 ± 2.09
Blood glucose on 120 min of OGTT (mmol/L)	119	5.86 ± 1.62	74	5.70 ± 1.45	81	6.06 ± 1.54
Fasting IRI (mU/L)	86	18.24 ± 9.86^∧∧∧^	72	8.16 ± 4.22***	69	20.35 ± 11.67
IRI on 60 min of OGTT (mU/L)	54	108.70 ± 78.26^∧∧^	62	65.50 ± 49.05***	54	129.26 ± 79.32
IRI on 120 min of OGTT (mU/L)	57	66.69 ± 50.11^∧^	61	39.40 ± 33.11***	53	90.22 ± 74.22
HOMA index	86	3.80 ± 2.41^∧∧∧^	72	1.56 ± 0.97***	68	4.32 ± 2.69
Fasting glucose/insulin ratio	86	0.34 ± 0.22^∧∧∧^	72	0.68 ± 0.39***	68	0.32 ± 0.22
Dislipidemia (%)	125	44.0%^∧∧∧^	91	15.4%***	81	40.7%
Treatment for dislipidemia (%)	125	4.8%	91	1.1%	80	2.5%
Total cholesterol (mmol/L)	122	4.73 ± 0.94	89	4.60 ± 0.97	81	4.68 ± 0.81
HDL cholesterol (mmol/L)	95	1.27 ± 0.28^∧∧∧^	52	1.52 ± 0.40**	66	1.28 ± 0.34
LDL cholesterol (mmol/L)	95	2.73 ± 0.88	52	2.78 ± 0.94	65	2.64 ± 0.73
VLDL cholesterol (mmol/L)	94	0.67 ± 0.43^∧∧∧^	49	0.37 ± 0.18***	64	0.71 ± 0.43
Triglycerides (mmol/L)	125	1.76 ± 0.75^∧∧∧^	89	0.91 ± 0.37***	80	1.56 ± 0.72
Arterial hypertension (%)	125	31.2%^∧∧∧^	93	1.1%***	81	29.6%
Treatment for arterial hypertention (%)	125	12.8%^∧∧^	93	1.1%***	81	18.5%
Systolic blood pressure (mmHg)	125	124.00 ± 12.44^∧∧∧^	90	113.78 ± 12.09***	81	123.77 ± 14.41
Diastolic blood pressure (mmHg)	125	80.00 ± 9.20^∧∧∧^	90	73.11 ± 8.16***	81	80.06 ± 10.29
cQT-interval (sec)	19	0.41 ± 0.02	9	0.41 ± 0.04	18	0.40 ± 0.03
RR-interval (sec)	22	0.73 ± 0.13^∧∧^	9	0.89 ± 0.16*	19	0.74 ± 0.09
Heart rate (bpm)	27	84.48 ± 11.46^∧∧∧^	9	68.22 ± 9.64*	25	79.48 ± 8.95

*P < 0.05; **P < 0.01; ***P < 0.001 between group 2 and group 3.

^*∧*^ *P* < 0.05; ^*∧∧*^ *P* < 0.01; ^*∧∧∧*^ *P* < 0.001 between group 1 and group 2.

*n* = number of patients.

**Table 3:** Comparison between liver enzymes and the rate of liver steatosis and the blood count between the three groups.

	Group 1		Group 2		Group 3	
	Obese		Lean PCOS		Obese PCOS	
	*n*		*n*		*n*	
ASAT (U/l)	100	20.08 ± 10.57	57	20.37 ± 21.10	66	25.27 ± 19.15
ALAT (U/l)	107	23.76 ± 16.33^∧∧^	61	17.57 ± 9.65**	71	28.07 ± 23.68
AP (U/l)	105	109.20 ± 46.102	54	90.85 ± 49.74**	62	120.58 ± 55.07
GGT (U/l)	101	24.11 ± 14.33^∧^	56	16.97 ± 11.62***	71	30.30 ± 21.90
Steatosis (%)	35	57.1%^∧∧^	13	7.7%***	25	72.0%
RBC	125	4.59 ± 0.49	89	4.51 ± 0.37	79	4.66 ± 0.39
Hemoglobin	125	135.58 ± 12.45	89	135.66 ± 12.40	80	139.80 ± 9.66^#^
Hematocrite	104	0.40 ± 0.04	86	0.40 ± 0.03	74	0.41 ± 0.03
MCV	88	86.05 ± 7.12^∧∧∧^	67	90.26 ± 4.96	56	87.77 ± 5.09
MCH	87	29.04 ± 2.78^∧∧∧^	67	30.48 ± 1.66	56	29.66 ± 1.71
MCHC	82	334.87 ± 17.14	63	336.83 ± 13.95	49	334.82 ± 19.79
Platelets	106	306.96 ± 82.36^∧∧^	81	272.69 ± 57.37	74	307.32 ± 67.85
MPV	78	8.58 ± 1.13	59	8.71 ± 0.98	46	8.73 ± 1.09
WBC	123	7.87 ± 2.51^∧∧^	87	6.88 ± 2.14*	80	7.84 ± 2.24
ESR	124	15.11 ± 9.16^∧∧∧^	83	7.75 ± 5.51***	78	14.38 ± 10.04

^#^ *P* < 0.05; between group 1and group 3.

*P < 0.05; **P < 0.01; ***P < 0.001 between group 2 and group 3.

^*∧*^ *P* < 0.05; ^*∧∧*^ *P* < 0.01; ^*∧∧∧*^ *P* < 0.001 between group 1 and group 2.

*n* = number of patients.

**Table 4:** Comparison of hormone levels and the indices of ovarian function between the three groups.

	Group 1		Group 2		Group 3	
	Obese		Lean PCOS		Obese PCOS	
	*n*		*n*		*n*	
Testosterone (nmol/L)	53	2.03 ± 1.22	85	2.42 ± 1.51	67	2.94 ± 1.72^##^
Estradiol (pmol/L)	37	0.39 ± 0.34	62	0.45 ± 0.47	40	0.72 ± 1.50
Androstendione (nmol/L)	17	11.58 ± 9.32	48	13.16 ± 9.33	33	13.34 ± 7.53
DHEAS (mcmol/L)	24	7.32 ± 4.29	60	9.24 ± 4.76	54	9.49 ± 4.98
17-OH-progesteron (nmol/L)	16	2.50 ± 1.45	37	3.40 ± 2.93	38	3.49 ± 2.76
LH (U/L)	42	2.91 ± 1.85^∧∧∧^	74	5.74 ± 4.01	59	5.17 ± 4.25^##^
FSH (U/L)	42	3.33 ± 1.94	76	3.40 ± 2.07	60	3.25 ± 1.81
Prolactin (U/L)	47	355.19 ± 235.88	54	388.45 ± 241.52	49	310.65 ± 184.77
Cortisol 8 : 00 o'clock (nmol/L)	52	405.14 ± 119.38	30	414.67 ± 156.03	41	392.35 ± 134.26
Cortisol 22:00 o'clock (nmol/L)	44	103.35 ± 97.67	15	111.37 ± 39.19	29	138.44 ± 113.19
TSH (U/L)	62	2.09 ± 0.99	39	2.02 ± 1.51	45	1.84 ± 1.17
Presence of polycystic ovaries (PCO) (%)	31	16.1%^∧∧∧^	85	84.7%	62	79.0%^###^
Right ovary PCO (%)	29	17.2%^∧∧∧^	74	87.8%	56	67.9% ^###^
Left ovary PCO (%)	29	13.8%^∧∧∧^	77	74.0%	56	75.0%^###^
Right ovary volume (mL)	18	6.99 ± 3.36^∧∧^	54	11.75 ± 6.16	37	10.37 ± 5.94
Left ovary volume (mL)	20	4.87 ± 3.06^∧∧^	54	9.18 ± 4.46***	35	11.13 ± 6.16
Age of menarche (years)	87	12.73 ± 1.66	86	13.05 ± 1.74	69	12.66 ± 1.62
Length of menstrual cycle (days)	44	29.55 ± 6.01^∧∧∧^	56	51.14 ± 23.12*	30	40.87 ± 16.86^##^
Duration of menstrual bleeding (days)	33	4.88 ± 2.84	44	4.95 ± 1.09	22	6.27 ± 5.55
Rate of menstrual disturbances (%)	107	39.3%^∧∧∧^	92	78.3%	79	81.0%^###^
Regular menstrual cycle (%)	106	65.1%^∧∧∧^	93	23.7%	80	20.0%^###^
Oligiomenorrhea (%)	104	28.8%^∧∧∧^	93	66.7%	80	66.2%^###^
Hypermenorrhea (%)	103	6.8%	91	3.3%	80	7.5%
Polymenorrhea (%)	103	2.9%	92	0%	80	1.2%
Opsomenorrhea (%)	103	3.9%	92	3.3%	80	2.5%
Amenorrhea (%)	103	5.8%	92	10.9%	80	13.8%
Pregnancies	98	0.59 ± 0.77^∧∧∧^	87	0.15 ± 0.45	81	0.42 ± 0.78
Births	97	0.51 ± 0.71^∧∧∧^	87	0.10 ± 0.34*	81	0.31 ± 0.63
Miscarriages	89	0.07 ± 0.36	86	0.05 ± 0.26	78	0.08 ± 0.35
Rate of infertility (%)	40	25.0%^∧^	20	55.0%	28	50.0%

^##^ *P* < 0.01;^ ###^ *P* < 0.001 between group 1 and group 3.

*P < 0.05; **P < 0.01; ***P < 0.001 between group 2 and group 3.

^*∧*^ *P* < 0.05; ^*∧∧*^ *P* < 0.01; ^*∧∧∧*^ *P* < 0.001 between group 1 and group 2.

*n* = number of patients.

**Table 5:** Comparison between the family history for chronic diseases between the three groups.

	Group 1		Group 2		Group 3	
	Obese		Lean PCOS		Obese PCOS	
	*n*		*n*		*n*	
Diabetes mellitus (%)	106	40.6%^∧^	79	24.1%*	75	42.7%
Arterial hypertension (%)	106	38.7%^∧∧^	79	19.0%*	75	37.3%
Dislipidema (%)	105	0.9%	79	0%	75	0%
Obesity (%)	106	28.3%^∧∧∧^	79	1.3%***	75	25.3%
CHD (coronary heart disease) (%)	106	0.9%	79	1.3%	75	5.3%
Menstrual disturbances (%)	106	0.9%	79	3.8%	75	1.3%
PCOS (%)	106	0%^∧∧∧^	79	12.7%*	74	2.7%

*P < 0.05; **P < 0.01; ***P < 0.001 between group 2 and group 3.

^*∧*^ *P* < 0.05; ^*∧∧*^ *P* < 0.01; ^*∧∧∧*^ *P* < 0.001 between group 1 and group 2.

*n* = number of patients.

**Table 6:** Comparison between the data about clinical hyperandrogenism between the three groups.

	Group 1		Group 2		Group 3	
	Obese		Lean PCOS		Obese PCOS	
	*n*		*n*		*n*	
Clinical hyperandrogenism	60	53.3%^∧∧^	83	77.1%***	72	86.1%
Hirsutism	60	51.7%^∧^	82	72.0%***	72	84.7%
Increased hair growth on the upper lip	48	31.2%^∧^	67	53.7%**	55	58.2%
Increased hair growth on the sideburns	46	23.9%^∧^	67	47.8%**	55	58.2%
Increased hair growth on the beard	47	37.6%	67	50.7%***	55	70.9%^#^
Increased hair growth on the chest	47	10.6%^∧∧^	67	34.3%**	54	37.0%
Increased hair growth on the abdomen	48	27.1%^∧∧∧^	67	62.7%**	56	60.7%
Increased hair growth on the inner thighs	48	14.6%^∧∧∧^	66	50.0%***	55	65.5%
Acne	50	4.0%	70	12.9%	62	1.6%
Androgenic alopecia	50	4.0%	73	6.8%	63	1.6%
Ferriman-Gallwey score	12	7.42 ± 2.47	37	9.16 ± 3.59	15	9.40 ± 4.17

^#^ *P* < 0.05; between group 1 and group 3.

*P < 0.05; **P < 0.01; ***P < 0.001 between group 2 and group 3.

^*∧*^ *P* < 0.05; ^*∧∧*^ *P* < 0.01; ^*∧∧∧*^ *P* < 0.001 between group 1 and group 2.

*n* = number of patients.

**Table 7:** Comparison between the reasons for admittance and the type of treatment before and after the admittance between the three groups.

	Group 1		Group 2		Group 3	
	Obese		Lean PCOS		Obese PCOS	
	*n*		*n*		*n*	
Hirsutism	125	4.0%^∧∧∧^	92	42.4%	79	35.4%^###^
Menstrual disturbances	125	9.6%^∧∧∧^	92	75.0%**	79	49.4%^###^
Infertility	125	4.0%	92	4.3%	79	8.9%
Obesity	125	72.0%^∧∧∧^	92	4.3%***	79	49.4%^##^
Other	124	20.2%^∧^	92	9.8%	79	7.6%^#^
Mean hospital stay (days)	125	8.42 ± 5.47^∧^	94	6.51 ± 4.37	82	7.89 ± 4.78
COC before the admittance	125	4.8%^∧∧∧^	92	46.7%***	78	34.6%
Metformin before the admittance	125	10.4%	92	7.6%	78	17.9%^#^
Combination of COC and metformin before the admittance	125	0%	92	0%**	65	7.7%^#^
Surgical treatment before the admittance	125	0%^∧∧^	92	6.5%**	78	7.7%
COC after the admittance	125	4.8%^∧∧∧^	92	28.3%***	78	28.2%
Metformin after the admittance	125	48.8%	92	44.6%***	78	78.2%^###^
Combination of COC and metformin before the admittance	125	1.6%^∧∧∧^	92	8.7%***	78	19.2%^#^
Surgical treatment after the admittance	125	0%	92	0%	78	0%

^#^ *P* < 0.05; ^##^ *P* < 0.01;^ ###^ *P* < 0.001 between group 1 and group 3.

*P < 0.05; **P < 0.01; ***P < 0.001 between group 2 and group 3.

^*∧*^ *P* < 0.05; ^*∧∧*^ *P* < 0.01; ^*∧∧∧*^ *P* < 0.001 between group 1 and group 2.

*n* = number of patients.

**Table 8:** Insulin resistance according to different criteria in the general group (*n* = 155).

	Number	% from the total number
IFG/IGT	20	12.9%
Increased baseline insulin	62	40.0%
Elevation of IRI over 100 mU/L on the 60 min of OGTT	62	40.0%
Elevation of IRI over 100 mU/L on the 120 min of OGTT	28	18.1%
Elevation of IRI over 100 mU/L on the 60 and/or 120 min of OGTT	66	42.6%
Elevation of IRI over 100 mU/L on the 60 and 120 min of OGTT	24	15.5%
Elevation of IRI more than 5 times over the baseline	108	69.7%
Decreased baseline glucose/insulin ratio (<0.333 mmol/L/mU/L)	71	45.8%
Increased HOMA-index (%)	93	60.0%

**Table 9:** Insulin resistance according to different criteria.

	Group 1		Group 2		Group 3	
	Obese		Lean PCOS		Obese PCOS	
	(*n* = 56)		(*n* = 56)		(*n* = 43)	
	*n*		*n*		*n*	
IFG/IGT	6	10.7%	5	8.9%	9	20.9%
Increased baseline insulin	28	50.0%	5	8.9%	29	67.4%
Elevation of IRI over 100 mU/L on the 60 min of OGTT	26	46.4%	9	16.1%	27	62.8%
Elevation of IRI over 100 mU/L on the 120 min of OGTT	12	21.4%	5	8.9%	11	25.5%
Elevation of IRI over 100 mU/L on the 60 and/or 120 min of OGTT	29	51.8%	10	17.9%	27	62.8%
Elevation of IRI over 100 mU/L on the 60 and 120 min of OGTT	9	16.1%	4	7.1%	11	25.6%
Elevation of IRI more than 5 times over the baseline (%)	39	69.6%	40	71.4%	29	67.4%
Decreased baseline glucose/insulin ratio (< 0.333 mmol/L/mU/L)	30	53.6%	12	21.4%	29	67.4%
Increased HOMA-index	39	69.6%	17	30.4%	37	86.0%
